# The characteristics of stroke and its rehabilitation in Northern Tanzania

**DOI:** 10.1080/16549716.2021.1927507

**Published:** 2021-08-03

**Authors:** Egfrid Michael Mkoba, Gunnevi Sundelin, Klas-Göran Sahlen, Ann Sörlin

**Affiliations:** a Department of Community Medicine and Rehabilitation, Physiotherapy, Umeå University, Umeå, Sweden; b Physiotherapy Department, Faculty of Rehabilitation Medicine, Kilimanjaro Christian Medical University College, Moshi, Tanzania; c Department of Epidemiology and Global Health, Umeå University, Umeå, Sweden

**Keywords:** Cardiovascular disease, low-income countries, rehabilitation, cross-sectional audit, stroke

## Abstract

**Background:**

Stroke causes great suffering and severe disability worldwide, and rehabilitation following a stroke seeks to restore lost functions. The extent to which stroke patients get access to rehabilitation in Tanzania is not well estimated, and drawing a current picture of the rehabilitation services for these persons is the first step in developing a more effective rehabilitation model in the country.

**Objective:**

The objective of this study was to establish the characteristics of stroke and its rehabilitation at the Kilimanjaro Christian Medical Centre (KCMC), a consultant referral hospital in northern Tanzania.

**Methods:**

This was a records-based descriptive study in which demographic, clinical, and rehabilitation information of stroke patients admitted to the KCMC between January 2012 and December 2015 was collected and audited. The means, percentages, and proportions were used to summarise the demographic, clinical, and rehabilitation patterns using SPSS version 24.0 software. The chi-squared statistic was used to examine the relationships between categorical variables, and a *p*-value<0.05 was considered statistically significant.

**Results:**

Of the 17,975 patients admitted to the KCMC during the period of the study, 753 (4.2%) had suffered a stroke, with a mean age of 68.8 ± 16.4 years. The predominant cause of stroke was hypertension, which accounted for 546 (72.5%) patients. A total of 357 (47.4%) patients had various forms of rehabilitation during the admission to hospital. Following a discharge home 240 (31.9%) patients did not return to the hospital for the continuation of rehabilitation.

**Conclusion:**

Stroke patients at the KCMC lack access to rehabilitation therapies. Insufficient access to rehabilitation therapies may warrant the need to explore alternative approaches such as tele-rehabilitation technologies in Tanzania.

## Background

Stroke causes great suffering and severe disability worldwide, and it is the third leading cause of premature disability as measured in disability-adjusted life years [1,2]. Stroke is the second most-common cause of death worldwide, causing approximately 5.8 million deaths every year, which is nearly 34.1% of the total deaths from cardiovascular diseases [3,4]. The incidence of stroke in high-income countries varies between 94.6/100,000 and 141.3/100,000 cases per year [5–7]. Published stroke incidence data with methodologically reliable designs are limited in low- and middle-income countries (LMICs), but the incidence has been estimated to range between 350/100,000 and 2120/100,000 cases per year [8]. In sub-Saharan Africa (SSA) the incidence ranges between 15/100,000 and 1460/100,000, but this range might be greater if more and better quality studies were to be performed [9,10]. However, it is difficult to conduct community-based studies in SSA because of the lack of resources and because many people do not have contact with medical services. Thus, almost all of the few existing studies in SSA are hospital based and thus reflect only those people who manage to reach a hospital [9–13].

Rehabilitation after a stroke usually seeks to restore lost functions to as normal a level as possible. In high-income countries, it is recommended to immediately commence rehabilitation during the admission to hospital followed by patient transfer to intermediate facilities for the continuation of rehabilitation [14]. This option is almost non-existent in LMICs, particularly in SSA [15–20]. Instead, community-based rehabilitation (CBR) remains the most efficient option for attending to the unmet needs of people with disabilities in SSA [21]. There are limitations, however, regarding CBR’s efficiency for the rehabilitation of stroke patients. For example, a study conducted in Ghana [22] showed that CBR was able to identify stroke patients but could not provide the resources or direction to significantly influence the care that was needed. In Tanzania, one study [23] showed that CBR served primarily children with neurological, visual, and/or auditory disabilities, and figures on stroke were not available. Another study in South Africa [24] showed that stroke patients received only limited rehabilitation therapies through CBR, which did not exceed five sessions over a period of six months. The rehabilitation of patients with various forms of disabilities has not been prioritised in SSA because governments focus primarily on the burden of communicable diseases [10,25], but the increasing rate of non-communicable diseases calls upon the countries of SSA to respond to the growing trends of these diseases [1,10,26].

Recently, Tanzania has reported an increase in deaths due to non-communicable diseases [27]. In 2015, stroke was for the first time listed among the major causes of death (ranked eighth) accounting for 3% of total deaths occurring in hospitals [28,29]. To the best of our understanding, the extent to which stroke patients in Tanzania are afflicted with stroke and subsequent disability is not known. While we know from Kingau [30] that the frequency of rehabilitation is a key determinant for recovery, the extent to which patients at the Kilimanjaro Christian Medical Centre (KCMC) get access to rehabilitation therapies during and after the admission to hospital is not known. However, stroke has been described as an important cause of admission to the KCMC.

The purpose of this study was to draw a characteristic picture of stroke and its rehabilitation at the KCMC, which might be the first step in identifying the extent to which stroke patients get access to rehabilitation. This might inform rehabilitation stakeholders regarding the lack of access to rehabilitation and thus lead to the development of more efficient approaches in this regard.

## Methods

### Design and setting

This is a records-based descriptive study in which demographic, clinical, and rehabilitation information on stroke patients admitted to the KCMC between January 2012 and December 2015 was collected and audited. The KCMC is a consultant teaching hospital in Moshi, a town in the Kilimanjaro region of northern Tanzania. It is among the top-ranked teaching hospitals in Tanzania with an in-patient capacity of 630, out of which 107 (16.9%) beds are in the medical ward where stroke patients are hospitalised. It has three rehabilitation-related departments set apart. These include physiotherapy, orthopaedic technology, and occupational therapy. The KCMC has no definitive rehabilitation structure. Thus, patients are attended separately as they are referred to the departments by medical practitioners. During the period of this study, there were ten physiotherapists; six occupational therapists; and six orthopaedic technologists. These staffs were available for all inpatients of all sorts of illnesses. Similarly, the same staffs were available for outpatients whose access to rehabilitation is characteristically influenced by socioeconomic and geographical factors, to mention a few.

Kilimanjaro is the smallest region in Tanzania with an area of 13,250 square kilometres. It has good roads, which can ease the transport of persons with movement difficulties. However, ambulances are scarce, and public and private transports are widely used to transfer patients, which is not always suitable for patients with movement difficulties. The study was approved by the research ethics committee of the KCMC, Moshi, Tanzania (ref. no. KCMC T.7/61).

### Data sources and collection

A data collection sheet was developed to collect demographic, clinical, and rehabilitation information. The name, age, sex, and place of domicile (urban/rural) of all patients admitted to the medical ward between 2012 and 2015 were obtained from the admission registers of the ward. A total of 753 stroke patients’ records were obtained from the register, and the list was submitted to the Health Records Department of the hospital for retrieval of each patient’s relevant clinical information. Demographic information included age, sex, marital status, and place of domicile. Clinical information included the date of stroke, date of admission to hospital, cause of stroke, sub-classification of stroke, outcome (dead/alive), and the date of discharge home. Rehabilitation information included the pattern of access to various forms of rehabilitation therapies, which was based solely on documented evidence of patient attendance and the number of documented contacts between the therapists and the patients.

### Inclusion and exclusion criteria

**Figure 1.**
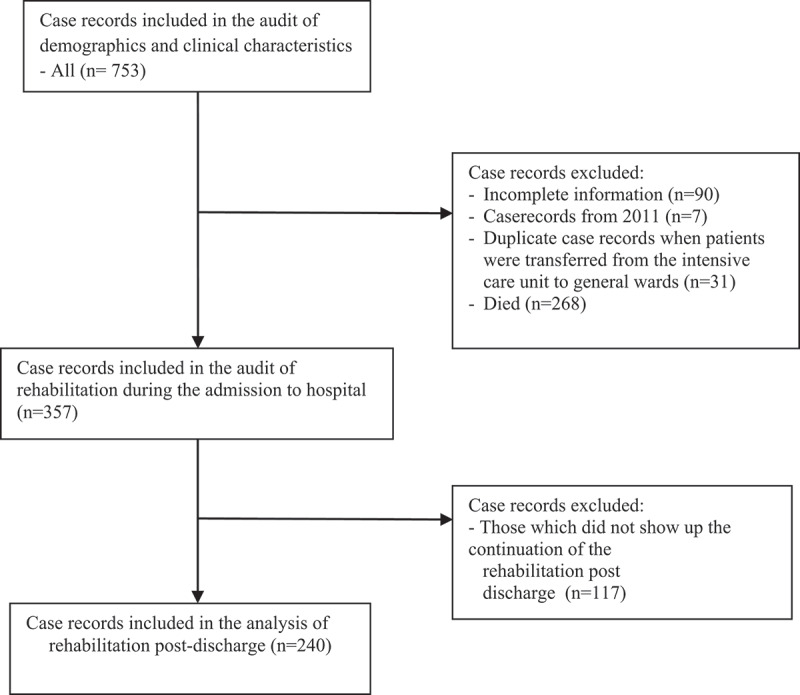
Study flow chart

All stroke case records obtained from the period of inquiry were included in the demographics and clinical characteristics aspects of the study. For the rehabilitation part of the study, all stroke records with ‘complete’ information and a diagnosis of stroke were included. Complete information here refers to those case records that had all the information necessary for the audit of rehabilitation of stroke patients. All case records that could not be retrieved, case records from years outside the inquiry period, and duplicate case records that appeared during patients’ transfer from the intensive care unit to the general ward were excluded (Figure 1).

### Statistical audit

Prior to the audit of the collected data, age-group categories (years) were constructed according to Tanzania’s age structure [28] as children (0–14 years), early working age (15–24 years), prime working age (25–54 years), mature working age (55–64 years), and elderly (≥65 years). None of the case records indicated any stroke patients among the children or early working age groups, and these age groups were excluded from the audit. Descriptive statistics of means, percentages, and proportions were used to summarise the demographic, clinical, and rehabilitation data using the software package SPSS version 24.0. The chi-squared statistic was used to examine relationships between categorical variables where a *p*-value <0.05 was considered statistically significant. Hospital length of stay was calculated using the admission and discharge dates, and the average length of stay (ALOS) for stroke patients was calculated as the total number of in-patients bed days (HBD) divided by the total number of admissions (discharges and deaths) (DC+D), thus HBD/(DC + D) = ALOS.

## Results

### Patient demographics and clinical characteristics

A total of 17,975 patients were admitted to the medical ward of the KCMC between January 2012 and December 2015, of whom 753 (4.2%) were stroke patients. Of these, 388 (51.5%) were males and 365 (48.5%) were females giving a male to female ratio of 1:1.06. The distribution between sex and place of domicile was not statistically significant (*p* > 0.05). The mean age for the occurrence of stroke for this group of patients was 68.8 ± 16.4 years.

The overall, in-hospital mortality following admission for stroke was 290 (38.5%), which predominantly occurred in males (163, 21.6%) compared to females (127, 16.9%). The majority of deaths (159 (21.1%)) occurred within the first week of the admission to hospital, of whom 70 (9.3%) died within the first 24 hours. The distribution of deaths according to sex, age, or place of domicile was not statistically significant (*p* > 0.05). The case fatality rate was highest during the first week at 31.7% and reached 39.5% at the end of one month. The ALOS during the admission to hospital was 10.2 days (95% CI 7.1–13.3 days). A total of 463 (61.5%) patients survived the stroke.

The predominant cause of stroke was hypertension, which accounted for 546 (72.5%) patients. A total of 145 (19.3%) patients had a combination of either HIV or diabetes with hypertension. Causes of stroke between age groups and sex were not statistically significant (*p* > 0.05). A total of 483 (64.1%) records showed that computed tomography (CT) scans had been requested by the physicians, but only 116 (15.4%) had been performed. Of these, 66 (8.8%) were sub-classified as haemorrhagic stroke and 39 (5.2%) were sub-classified as ischaemic stroke. The majority of the patients (479 (63.6%)) were ≥65 years old. Most stroke patients (488 (64.8%)) had left side paralysis.

**Table1.** Mortality of stroke cases by age group categories and sex (*n* = 753)

Age group categories	Sex	Alive	Dead	*p*-Value
Prime working age (25–54 years)	Female	56 (78.9)	15 (21.1)	0.002*
	Male	43 (55.8)	34 (44.2)	
	Total	99 (69.9)	49 (33.1)	
Mature working age (55–64 years)	Female	45 (78.9)	12 (21.1)	0.004*
	Male	38 (55.1)	31 (44.9)	
	Total	83 (65.9)	43 (34.1)	
Elderly (≥65 years)	Female	137 (57.8)	100 (42.2)	0.388
	Male	144 (59.5)	98 (40.5)	
	Total	281 (58.7)	198 (41.3)	
Total	Female	238 (65.2)	127 (34.8)	0.025*
	Male	225 (58.0)	163 (42.0)	
	Total	463 (61.5)	290 (38.5)	

*Statistically significant.

**Table2.** Causes of stroke by sex and age group categories (*n* = 753)

Age group categories	Sex	Not Mentioned	Hypertension	Hypertension/diabetes & HIV	*p*-Value
Prime working age (25–54 years)	Female	5 (7.0)	54 (76.1)	12 (16.9)	0.745
	Male	7 (9.1)	60 (77.9)	10 (13.0)	
	Total	12(8.1)	114 (77.0)	22 (14.9)	
Mature working age (55–64 years)	Female	5 (8.8)	42 (73.7)	10 (17.5)	0.307
	Male	8 (11.6)	42 (60.9)	19 (27.5)	
	Total	13(10.3)	84 (66.7)	29 (23.0)	
Elderly (≥65 years)	Female	19 (8.0)	172 (72.6)	46 (19.4)	0.969
	Male	18 (7.4)	176 (72.7)	48 (19.8)	
	Total	37(7.7)	348 (72.7)	94 (19.6)	
Total	Female	29 (7.9)	268 (73.4)	68 (18.6)	0.862
	Male	33 (8.5)	278 (71.6)	77 (19.8)	
	Total	62 (8.2)	546 (72.5)	145 (19.3)	

### Rehabilitation during the admission to hospital

A total of 357 patients had access to various forms of rehabilitation therapies. Less than 50% of them had three or more sessions per week. There was no significant difference between sex, age groups, or place of domicile in terms of being provided with rehabilitation therapies (*p* > 0.05).

**Table3.** Inpatient rehabilitation session frequencies by sex, age group categories, and place of domicile (*n* = 357)

	Frequency of rehabilitation sessions, *n* (%)			
Variable categories	1 time	2 times	≥3 times	*p*-Value
Sex				
Female	30 (47.6)	70 (51.1)	80 (51.0)	0.887
Male	33 (2.4)	67 (48.9)	77 (49.0)	
Total	63 (17.6)	137 (38.4)	157 (44.0)	
Age group categories				
Prime working age (25–54 years)	14 (22.2)	24 (17.5)	40 (25.5)	0.542
Mature working age (55–64 years)	10 (15.9)	26 (19.0)	29 (18.5)	
Elderly (≥65 years)	39 (61.9)	87 (63.5)	88 (56.1)	
Total	63 (17.6)	137 (38.4)	157 (44.0)	
Place of domicile				
Urban	24 (38.1)	45 (32.8)	63 (40.1)	0.426
Rural	39 (61.9)	92 (67.2)	94 (59.9)	
Total	63 (17.6)	137 (38.4)	157 (44.0)	

### Rehabilitation post-discharge

A total of 240 patients returned to the KCMC for the continuation of rehabilitation as outpatients. The majority of them (95 (22.1%)) received three rehabilitation sessions per week, making the rehabilitation frequency of 2.3 sessions per week. Age groups and place of domicile significantly influenced the rehabilitation attendance frequency (*p* < 0.05).

**Table4.** Post-discharge rehabilitation session rates by sex, age group categories, and place of domicile (*n* = 240)

	Frequency of rehabilitation sessions, *n* (%)			
Variable category	1 time	2 times	≥3 times	*p*-Value
Sex				
Female	27 (22.3)	41 (33.9)	53 (43.8)	0.402
Male	31 (26.1)	46 (38.7)	42 (35.3)	
Total	58 (24.2)	87 (36.2)	95 (39.6)	
Age group categories				
Prime working age (25–54 years)	7 (10.6)	16 (24.2)	43 (65.2)	<0.001
Mature working age (55–64 years)	7 (13.2)	24 (45.3)	22 (41.5)	
Elderly (≥65 years)	44 (36.4)	47 (38.8)	30 (24.8)	
Total	58 (24.2)	87 (36.2)	95 (39.6)	
Place of domicile				
Urban	15 (12.8)	28 (23.9)	74 (63.2)	<0.001
Rural	43 (35.0)	59 (48.0)	21 (17.1)	
Total	58 (24.2)	87 (36.2)	95 (36.9)	

## Discussion

The purpose of this study was to draw a characteristic picture of stroke and its rehabilitation at the KCMC. During the period of this study, stroke was the fourth most common cause of death, accounting for roughly one-tenth of the total deaths in the medical ward. The mean age of stroke was higher than previously reported [9,11,27,28]. We conclude from this that many elderly patients were admitted to hospital earlier than the other age groups because they were sick enough to require immediate medical attention. Similar findings have been reported by Nakiibuka et al. [31] showing that only seriously ill patients are immediately brought to hospitals. This could be one reason for the tendency of mean age of stroke in patients admitted to hospitals to be higher. Ironically, those who are not seriously ill despite apparent inability to perform tasks may not immediately go or be taken to hospital.

The sub-classification of stroke did not seem to be essential in terms of the management of the stroke as more than a third of the patients were clinically reported as having stroke secondary to hypertension. This was probably because diagnosing stroke using CT scans was rare. More than half of the patients’ records showed that physicians had requested CT scan investigations, but less than a quarter of the requests were performed. Clinical examination alone is not reliable for the differentiation of stroke sub-types and thus for the proper management of stroke. It is probable, therefore, that some patients did not receive the appropriate treatment because of improper diagnoses. Lekoubou, Ekeh, and Tungu [32–34] showed that the inability to afford CT scan investigations in LMICs is due to limited access to health insurance and the inability of patients to pay out of pocket. This situation will continue unless appropriate response to the problem is considered. It is worth noting that there are increasing efforts to educate people in Tanzania on the benefits of health insurance packages. This may change the situation in the future.

The leading cause of stroke was hypertension, and some patients also had diabetes or HIV infection. Nearly one-tenth of the patients whose clinical records did not indicate the cause of their stroke were also likely to be hypertensive because hypertension has been shown to be an important risk factor for stroke in SSA [4,11,22,32,35]. The low level of HIV infection in this study might be because the physicians treated the stroke without testing for co-morbidities. This presumption is comparable to the findings of Kaseke et al. [13] in which only those patients admitted to hospitals in Zimbabwe with indications for HIV were tested for the virus. It is likely also that the number of patients with HIV infection in our study would have been larger had all patients been tested. The scale of HIV infection in our study population (19.3%) partly agrees with previous reports from the KCMC and Muhimbili National Hospital [36,37] where the rate of HIV infection among patients presenting with stroke was 20% and 20.9%, respectively.

Nearly half of the elderly patients died, which is not surprising because the likelihood of death following a stroke increases with age. Half of the prime working age and a third of the mature working age groups also died. The number of females in the prime working age and the elderly groups who died was significantly greater compared to the mature working age group. This is opposite to the trend in males in which death declined steadily with age. This might suggest that the males in this cohort lived longer after a stroke than females. Generally, the distribution of the attributes in this study group supports the assumption that stroke in SSA affects people equally regardless of their differences in sex or place of domicile.

### Rehabilitation during the admission to hospital

About three quarters of the patients had accessed rehabilitation therapies during the period of admission to the hospital, and the majority of those who accessed the service received three sessions per week. Owing to the fact that the ALOS was roughly 10 days, none of them had received more than six sessions. Shortage of staff for rehabilitation is one among the reasons for the limited access to rehabilitation by the patients. The medical ward admits all patients related to internal medicine, and the rehabilitation staff particularly physiotherapists working in the ward are required to serve all patients in need of the rehabilitation. It is likely that the burden of work among physiotherapists was huge enough to make them unable to cover the entire population of the stroke patients. The National Institute for Health and Care Excellence (NICE) [38] recommends a minimum frequency of five days of rehabilitation per week. This is just ‘every day’ over the weekly working days, which might also be suitable for the KCMC.

About a quarter of the patients did not get access to rehabilitation therapies. In practice, rehabilitation in Tanzania is not usually accessed without a physician’s referral. This is another reason for physiotherapists’ inability to reach all stroke patients during the admission to hospital. We believe that the physicians delayed referring the patients for rehabilitation because they were seriously ill. Previous authors [39,40] have shown that life-threatening problems in stroke vary in severity among patients and practice settings and that such problems may influence physicians’ decisions regarding the initiation of rehabilitation. Kingau [30] also showed that some patients admitted to hospitals in Kenya received physiotherapy one week later after their conditions had stabilised. These studies altogether suggest that the patient’s current health state is a good predictor of rehabilitation onset during the admission to hospital. Passive movements in the early stage, for example, can improve the function of upper extremities and activities of daily living. These are a part of early rehabilitation and do not usually cause unfavourable outcomes in stroke patients [40]. Such explanations may inform medical practice on the need to start rehabilitation immediately in the future regardless of the current state of the patient.

### Rehabilitation post-discharge

About one-fifth of the patients did not return to the hospital for the continuation of the rehabilitation post-discharge . We could identify no reason for why these patients did not come. However, some patients undoubtedly had died, and others decided perhaps not to continue with the rehabilitation because of reasons reported by previous authors. These include, to mention a few, socioeconomic, stroke-related stigma and limited geographic access to rehabilitation [24,35,41,42]. It is also likely that some patients were not discharged through rehabilitation departments; thus, they had thought that the rehabilitation had ended. For example, one study in the Gambia [43] showed that some patients had access to physiotherapy during the admission to hospital but on discharge home they were put on antihypertensive drugs without any rehabilitation plan. A similar picture may have also appeared at the KCMC.

Reports show those patients who continued with the rehabilitation progressively reduced their attendance to an average of two sessions per week over three successive weeks. This was followed by attending inconsistently to 12^th^ week where we could no longer trace the pattern of the attendance. This means that the majority of the patients had substantially accessed rehabilitation for the minimum duration of rehabilitation of 12 weeks while they should have successively access the service for up to 24 weeks. This timeframe is in accordance with evidence-based stroke rehabilitation care described by Bernhardt et al. [14]. We see here a progressive decline in follow-up after a discharge home, which is not favourable for patients. Olaleye and Platz [39,44] have described the lack of personnel to cause negative influence on patients’ motivation to attend rehabilitation. Ogwumike et al. [45] found that compliance to treatment was strongly influenced by workplace limitations, treatment dissatisfaction, lengthy waiting times, and inconvenient treatment times, and all of these could be causes for the poor attendance among patients in the present study. We believe that such shortcomings in accessing rehabilitation therapies at KCMC particularly at post-discharge would be partly eased by CBR programme. However, these centres are scarce in Tanzania. A study conducted in Tanzania [46] identified only 11 CBR centres whose staff body among others were only seven physiotherapists, six occupational therapists and three orthopaedic technologists. These numbers means that a few centres had no staffs for the rehabilitation. Lack of staff for rehabilitation in Tanzania has been reported [47] and this may be one of the causes that render the centres to conduct two major activities – rehabilitating children with disabilities through teaching their mothers/caretakers on handling and identifying persons with disabilities including stroke patients and referring them to hospitals for expertise care and rehabilitation.

In view of meeting the rehabilitation needs for stroke, the necessary healthcare structure for the rehabilitation of stroke in Tanzania is practically non-existent. We recognise the work done by Giglioni, Bonaventure, Mwinuka and Swai [46] attempting to develop path to the implementation of the Disability Act in Tanzania. Yet the development of guidelines for the rehabilitation needs immediate attention. The lack of personnel for rehabilitation needs to be strongly addressed, and where possible home-based rehabilitation using the developed guidelines should be put in place. A well-structured home-based rehabilitation may increase coverage of stroke patients and therapy time, which may attribute to patients improvement. Study by Edgar et al. [42] showed a positive relationship between increased amounts of therapy time and improvement in stroke rehabilitation. Recent pilot studies [35,41] on the use of telerehabilitation technology as an adjunct to home-based rehabilitation to improve outcomes after stroke in Ghana and Uganda have shown promising results. This technology can as well be piloted in Tanzania.

The following are the key findings of this study:

The characteristic picture of stroke in KCMC shows elderly persons (≥65 years) are more affected by stroke (281 (58.7%)) compared with mature working age (55–64 years) group (83 (65.9%)) and prime working age (25–54 yrs) group (99 (69.9%)). Males (388 (51.5%)) are more affected than females (365 (48.5%)) although not statistically significant. The stroke is predominantly caused by hypertension, and the overall in-hospital stroke mortality is 38.5%.

- Approximately one-third of stroke patients admitted to the hospital do not get access to rehabilitation. Similarly, one-third of patients do not get access to rehabilitation post-discharge.
- The frequencies of rehabilitation for those who get access to rehabilitation during the admission to the hospital and at post-discharge are lower than recommended.
- The active rehabilitation duration for stroke patients at the KCMC does not exceed 12 weeks from the time of admission to the hospital.

## Conclusion

Access to rehabilitation for stroke patients at the KCMC is influenced by complex relationships between its tentative rehabilitation structure and patients’ factors. Our findings suggest that interventions that address these modifiable aspects might improve access to rehabilitation. Telerehabilitation technology may improve access to, and outcomes of rehabilitation.

## Strengths and limitations of this study

The main strength of this study was that it is the first of its kind in Tanzania to report on a characteristic picture of stroke and its rehabilitation. However, it used data from patient files. Therefore, the subjective quality of the documentation of rehabilitation personnel might have had a negative influence on the results. In addition, patients whose incomplete records were excluded from the audit during hospitalisation and those may be considered here as not having access to the rehabilitation during hospitalization, but they may have died before having access to it. Correspondingly, those 117 (32.8%) who did not return to continue with the rehabilitation post-discharge may have died at home, lost interest to rehabilitation follow-up, strangled by variety of reasons as has been reported by previous authors or migrated to other communities making travel to the KCMC unnecessary. Such uncertain data may have additionally biased the findings.

## Paper context

Drawing a picture of access to rehabilitation for stroke patients is important in warranting the need to explore approaches for improved care. The extent to which stroke patients get access to rehabilitation therapies in Tanzania is not fully understood. Therefore, drawing a characteristic picture of rehabilitation of stroke patients in one of its tertiary hospitals is one step in envisaging improvement of rehabilitation services in the country.
